# The State of Extracorporeal Shockwave Therapy for Myofascial Pain Syndrome—A Scoping Review and a Call for Standardized Protocols

**DOI:** 10.3390/life15101501

**Published:** 2025-09-24

**Authors:** Hannes Müller-Ehrenberg, Jacopo Bonavita, Yunfeng Sun, Carla Stecco, Federico Giordani

**Affiliations:** 1 Private Clinic Orthopädische Privatpraxis, 48143 Münster, Germany; info@triggerpunktzentrum.de; 2 Villa Rosa Rehabilitation Hospital, APSS, 38057 Trento, Italy; 3 Department of Anatomy, University of Padova, 35122 Padova, Italy

**Keywords:** shock wave, shockwaves, myofascial pain, ESWT, myofascial trigger point, fascia, review, meta-analysis

## Abstract

Background: Extracorporeal Shockwave Therapy (ESWT) for targeting myofascial tissues is gaining increasing interest for the treatment of musculoskeletal disorders. This review evaluates the mechanisms, applications, and effectiveness of ESWT in managing myofascial pain syndrome (MPS) while identifying methodological gaps in existing research. Methods: A systematic search of PubMed, PEDro, and Cochrane Central Library was conducted up to August 2025, focusing on studies from existing meta-analyses, particularly randomized controlled trials. Eligible studies were selected based on predefined criteria, including the use of ESWT for MPS treatment, methodological rigor, and adherence to standardized protocols. Data were extracted on diagnostic criteria for MPS and myofascial trigger points (MTrPs), shockwave application parameters, adherence to International Society for Medical Shockwave Treatment (ISMST) guidelines, follow-up periods, and treatment efficacy. Results: significant inconsistencies were identified in MPS diagnosis, shockwave application technique, and study follow-up periods. Many studies did not adhere to ISMST guidelines, with variations in energy levels, impulses, and differentiation between radial pressure wave (RPW) and focused ESWT (fESWT). One-third of the studies had follow-up periods of two weeks or less, limiting the assessment of long-term outcomes. Despite these limitations, ESWT demonstrated moderate to good efficacy compared with controls. Conclusions: While ESWT appears effective for MPS, methodological inconsistencies prevent definitive conclusions. Future research should standardize protocols, differentiate RPW from fESWT, and include longer follow-up periods to optimize therapeutic potential and validate ESWT as a treatment for MPS.

## 1. Introduction

Myofascial tissue constitutes approximately 40–50% of the human body mass. Recent research indicates that numerous neurological structures capable of generating pain are present in muscles and fascia [1,2,3]. The symptoms and pain associated with myofascial tissue are collectively known as myofascial pain syndrome (MPS) or myofascial syndrome (ICD-10 M79.1). MPS is recognized as a common cause of musculoskeletal pain and can typically mimic conditions of articular dysfunction, neurological and orthopaedic diseases, which leads to significant clinical relevance [4,5,6].

Extracorporeal shockwave therapy (ESWT) is a standard procedure for the treatment of musculoskeletal disorders due to its ability to reduce pain and promote regenerative processes in the treated tissue [7,8]. Furthermore, ESWT has been established as a treatment modality for MPS [9,10]. In recent years, a substantial body of research has been conducted on the use of shockwaves for the treatment of myofascial pain syndrome (MPS). Additionally, numerous reviews and meta-analyses examined the development of myofascial ESWT [10,11,12,13,14,15,16]. The findings of these studies and reviews generally suggest that ESWT is an effective treatment for MPS. However, these outcomes are not substantially superior to those achieved through other conventional treatment modalities, including trigger point injection, dry needling, and laser therapy. This contrasts with the results of other studies, which indicate that ESWT is one of the most effective modalities for treating various musculoskeletal disorders, including tendinitis, plantar fasciitis, chronic pelvic pain, and rotator cuff disease [8,17,18]. It is therefore pertinent to question why myofascial ESWT, which treats muscles and fascia as target tissue, is not equally successful.

The primary aim of this scoping review is to provide an overview of the concept of myofascial syndrome treatment and the mechanisms of action of ESWT on muscle and connective tissue. Furthermore, this review will explain the results of the available data from the reviews and meta-analyses. The present scoping review is based on a detailed analysis of all studies selected for the present meta-analyses, conducted in accordance with standardized criteria. This analysis of content includes the examination protocols for MPS, the precision of shockwave application, and the follow-up.

### 1.1. Myofascial Pain Syndrome

MPS is a prevalent condition in the spectrum of musculoskeletal disorders, affecting between 21% and 93% of individuals with complaints of musculoskeletal pain [19,20,21]. It is characterized by the presence of myofascial trigger points (MTrPs) [4]. These are discrete, focal, hyperirritable spots located in a taut band of skeletal muscle. They cause pain directly and refer pain to distant sites. MPS is characterized by persistent regional pain, frequently accompanied by motor dysfunction and autonomic phenomena [4,5,22,23]. The etiology of MPS is multifactorial, involving mechanical, biochemical, and psychological components that contribute to the development and maintenance of MTrPs [4,5,6,22,23,24,25]. Primary factors include muscle overuse, injury, and postural dysfunctions, which lead to repetitive motions, sustained loading, eccentric muscle activity, and muscle fatigue. These factors can trigger the formation of MTrPs. Such conditions result in localized muscle tension and hypoxia, which are pivotal in the genesis of trigger points [6,22,24]. The diagnosis of MFS entails a comprehensive neurological–orthopaedic examination, with a particular focus on the examination according to the diagnostic criteria established by Travell and Simons [4]. The primary diagnostic criteria are “recognition” and “referral” of pain, which involve identifying muscles and fascia, as well as detecting myofascial trigger points through mechanical stimulation, mainly manual palpation [4,26,27,28]. Despite the development of diagnostic modalities for the diagnosis of MTrPs, including intramuscular needling, surface electromyography, infrared thermography, elastography, and ultrasound, these methods have yet to be accepted as reliable diagnostic methods [29,30]. In scientific studies, magnetic resonance imaging scans are employed, yet they have yet to be validated for clinical use [31]. Additionally, ultrasound examination with high-definition imaging and elastography are garnering attention for a more comprehensive understanding of myofascial tissue [29]. A variety of treatment modalities have been employed with the specific aim of treating MTrPs, including trigger point injection, dry needling, laser therapy, etc., as well as extracorporeal shockwave therapy, which has shown good results in the treatment of MFS [9,32,33,34,35,36,37,38].

### 1.2. Basics of Extracorporeal Shockwave Therapy

ESWT was first introduced 40 years ago for the therapeutic destruction of kidney stones (lithotripsy) [39]. Over the past three decades, ESWT has also been employed in the treatment of musculoskeletal disorders. Basic studies and clinical trials have shown that ESWT is a safe and effective method for treating various musculoskeletal diseases [7,8,17,18,38]. Initially, ESWT was primarily used to treat bone and calcified structures in orthopaedics [39,40]. However, recent studies have demonstrated the efficacy of ESWT in targeting other tissue types, including skin, nerves, and myofascial tissue, for medical intervention [7,9,11,41,42,43,44].

Two distinct types of energy are employed in the context of medical shockwaves: focused extracorporeal shockwave therapy (fESWT) and radial pressure waves (RPWs). These technologies diverge in their generation devices, physical characteristics, and mechanisms of action [45,46,47]. RPWs deliver most of their energy to the surface, after which it expands radially into the tissue. Notably, the physical characteristics of a shockwave are absent due to the prolonged rise times of pressure pulses and the relatively low-pressure outputs [47,48]. The primary drawback of radial pressure wave therapy is its limited depth of penetration and reduced efficacy in stimulating cellular processes [45,47]. The biological effects of RPW differ from those of focused shockwaves due to variations in the pressure waveform [45,46,47,48]. RPW is indicated for the treatment of superficial tissue, while fESWT can reach deeper tissue layers with concentrated energy [47,48]. fESWT is distinguished by its high peak pressure (up to more than 100 MPa or 500 bar), rapid pressure rise (less than 10 nanoseconds), brief duration (less than 10 nanoseconds), and broad range of frequencies [45,48]. fESWT is generated by electrohydraulic, piezoelectric, or electromagnetic generators [45,48]. When applied correctly in accordance with the International Society for Medical Shockwave Therapy (ISMST) guidelines, low- to medium energy level shockwaves (0.01–0.3 mJ/mm^2^) do not cause mechanical destruction of the musculoskeletal system. Instead, they affect tissue and cellular function and metabolism through a process known as mechanotransduction [7,18].

The following mechanisms of action are applicable to all types of tissue and are also involved in the regeneration of myofascial tissue:

- Angiogenesis through up-regulation of NO and VEGF [7,17,18,49,50,51].
- Mechanotransduction stimulating stem cells [52,53].
- Modulation of inflammation [41,54].
- Reduction of vasonociceptive-active substances (e.g., Substance P, CGRP) [55,56,57].

### 1.3. Specific Mechanisms of Action of ESWT on Myofascial Tissue

The progress in research on connective tissue histology and pathophysiology has expanded the definition of the fascial system to include tendons and intra- and intermuscular connective tissues [58]. Tendinous tissue, which is part of the fascial family, has been successfully treated with ESWT for 25 years [8,17,18]. A multitude of fundamental studies and clinical trials in this field have permitted the extrapolation of the effects of ESWT on all fascial tissues, not just tendons.

Fibroblasts are considered to be the major mechano-responsive cells in the connective tissue [59]. Responsible for organizing and synthesizing connective tissue, fibroblasts are essential for remodeling the extracellular matrix [60]. In vitro and in vivo studies have demonstrated that ESWT treatment enhances fibroblast proliferation and differentiation by activation of gene expression for transforming growth factor β1 (TGF-β1) and collagen types I and III [61,62]. Furthermore, recent research suggests that collagen cell production after fESWT is enhanced 24 and 48 h after stimulation [63]. Additionally, an increase in nitric oxide (NO) release has been reported during the early stages of treatment, and the subsequent activation of endothelial nitric oxide synthase (eNOS) and of vascular endothelial growth factor (VEGF) is related to TGF-β1 rise [61]. Furthermore, the increase in angiogenesis observed in ESW-treated tendons is an additional factor in accelerating the repair process [61].

Additionally, direct effects of ESWT on the extracellular matrix have been described [64]. It is therefore assumed that ESWT can positively modulate these actions with a beneficial effect on myofascial tissue regeneration and healing processes. Several in vivo and in vitro studies have confirmed an enhancement of fibroblast proliferation after ESWT [61,65]. Moreover, in the treatment of numerous diseases involving fibrous tissue (e.g., M. Ledderhose) it has been shown that focused ESWT reduces the fibrotic load by modulating the pro- and antifibrotic proteins TGF-β and MMP-2, resulting in an antifibrotic effect [42,66,67,68]. This antifibrotic effect can also be explained on a histopathological level, with ESWT down-regulating alpha-SMA expression, collagen type I, and myofibroblast phenotype [69,70]. Recent research has demonstrated the potential of ESWT to facilitate the correct gliding between myofascial layers by improving hyaluronic acid viscoelasticity [63]. It is crucial to deepen our understanding of the effects of ESWT on fasciacytes, which are specialized fibroblast-like cells in fasciae responsible for the biosynthesis of HA-rich matrices [71].

In fundamental research studies, it has been demonstrated that vasonociceptive-active substances involved in the inflammatory response, such as Substance P, COX-2, Prostaglandin-E2, CGRP, and others have been reduced by the application of shockwave energy [44,55,72]. The significant reduction of Substance P by ESWT application may lead to a further decrease in pain [55]. In vivo studies in humans have identified elevated levels of Substance P, CGRP, bradykinin, and other pain-related vasonociceptive-active substances in myofascial tissue, with a greater concentration observed in MTrP [73]. These findings can explain the positive effects observed in clinical studies, with the application of ESWT leading to a reduction in myofascial pain [11,12,14,15,74].

Since the late 1990s, ESWT has been directly applied to muscle tissue to reduce muscle tone in individuals with spasticity [75]. Some studies have demonstrated good outcomes [75,76]. fESWT treatment targeting muscular tissue has shown to have immediate effects on pain relief in acute muscle injuries and has provided evidence for accelerated regeneration of damaged skeletal muscle [68,77,78,79]. In recent years, myofascial tissue and, in particular, myofascial trigger points have emerged as a key focus of ESWT. Despite the absence of a widely accepted therapeutic protocol for myofascial shockwave therapy, numerous studies have been conducted in this area in recent years [9,11,15,74,80,81].

## 2. Materials and Methods

**Figure 1:**
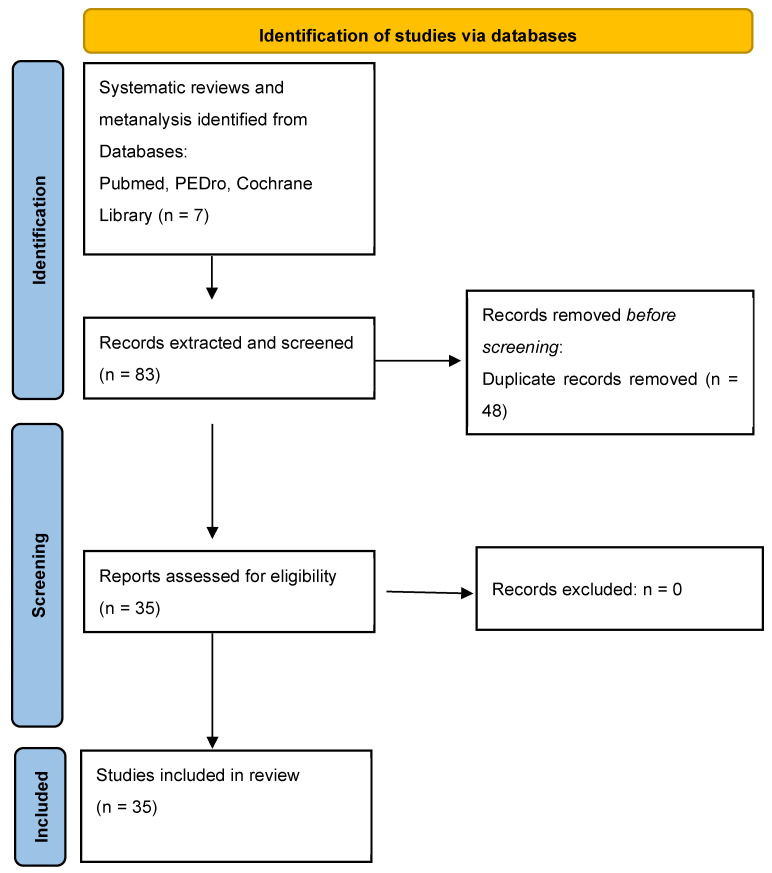
Flow diagram for study extraction, screening, and inclusion.

A bibliographic search was conducted in the scientific online database (PubMed, PEDro, and Cochrane Central Library) using the combination of keywords “Extracorporeal Shockwave Therapy”, “shockwave”, “shock wave”, “myofascial”, and “myofascial pain” within the meta-analysis and systematic review published up to 30 August 2025. The search for and extraction of papers were conducted by two independent authors. The studies reported in the selected systematic reviews and meta-analyses were considered for the subsequent analysis. The comprehensive search strategy is outlined in Figure 1. Following the removal of duplicate publications, data were extracted for each study by the following parameters: number of patients included, type of technology (fESWT vs RPW), examiner and operator experience and qualifications, number of sessions, interval between sessions, protocol parameters (intensity, frequency, number of impulses), anatomical region and structure targeted, diagnostic criteria for definition of MPS, criteria for myofascial trigger point identification, and follow-up duration.

## 3. Results

A total of 7 systematic reviews and meta-analyses were identified through the previously described search strategy [10,11,12,13,14,15], and 83 studies were extracted from the selected papers. Following the removal of duplicates, a total of 35 full-text articles were subjected to assessment [80,82,83,84,85,86,87,88,89,90,91,92,93,94,95,96,97,98,99,100,101,102,103,104,105,106,107,108,109,110,111,112,113,114,115]. The characteristics of the included studies are presented in Table A1 and Table A2.

Of the 35 studies included in the review, 23 employed RPW, while 12 utilized fESWT. MPS was diagnosed in 20 trials based on the Travell and Simons criteria [84,85,90,91,92,93,95,96,97,98,99,100,102,103,106,108,110,111,114,115]. In the remaining trials, patients were classified as having clinically diagnosed MPS, which is characterized by the presence of palpable taut bands, a painful spot (MTrP), or referred pain. Only six examinations documented the recognition of pain [80,89,102,108,111,113]. In one study treating latent MTrP [113], patients with this diagnostic criterion were explicitly excluded. Seventeen studies reported the examiner’s qualifications [83,84,85,86,92,96,97,102,103,106,107,109,110,112,113,114,115], and six documented the examiner’s experience (range: three to twenty years) [85,102,103,106,110]. In the remaining studies, no qualification or experience data were stated [80,82,87,88,89,90,91,93,94,95,98,99,100,101,104,105,108,111]. Only 14 of the 35 studies provided information regarding the background [83,84,85,87,92,96,102,103,106,107,109,112,114,115] and 4 the experience of the operator performing the intervention [85,103,106,114]. Additionally, 28 of 35 studies reported the criteria for MTrP identification, while 23 provided information regarding the diagnostic criteria that were employed during the application of shockwaves on the MTrP (Table A1 for reference). Four studies did not provide information regarding the targeted anatomical structure [84,94,101,110].

In terms of the protocols and parameters, the number of shocks per MTrP ranged from 300 to 2000. Seven studies did not provide any data regarding the number of impulses administered per point [80,87,92,99,101,111,112]. The total number of shocks administered per session ranged from 1000 to 24,000, while the energy flux density (EFD) ranged from 0.03 to 0.38 mJ/mm^2^ and from 0.12 to 6 bar for RPW. The frequency of treatment ranged from 1 Hz to 20 Hz, and 12 studies did not provide this information [80,86,87,91,92,93,96,97,102,104,106,115]. The number of sessions ranged from 1 to 30, with an interval of days between sessions ranging from three to seven. The total time of follow-ups ranged from immediate post-treatment to 15 weeks, with 13 studies having a final examination of two weeks or less [80,86,87,88,96,99,103,106,108,109,110,113,114]. One failed to document this information [100].

## 4. Discussion

This is the first study to examine the information provided in the selected publications regarding the initial examination for myofascial pain, the exact method of shockwave application, and the follow-up. Our review identified discrepancies among the studies included in the currently available meta-analyses and systematic reviews.

Initially, the data analysis of four reviews did not differentiate between RPW and fESWT [12,13,14,16]. Two reviews distinguished between RPW and fESWT [11,15], whereas one review included only studies employing fESWT [10]. Given the substantial differences between the two technologies, both in terms of the type of stimulus and the biological effect on tissues, it is essential to make a precise distinction to facilitate a comparison of results between independent studies. Radial pressure wave (RPW) displays marked differences in its physical characteristics when compared with focus shockwaves. RPW generates lower peak pressures, delivers the maximum energy at the point of application to the skin, and propagates outwards without a focal point. Consequently, fESWT is regarded as a more suitable modality for specific and deeper structures, offering precise stimulation.

Additionally, a lack of transparency in the diagnosis of MPS and the reporting of MTrP diagnostic criteria has been identified in the specialist literature. The available studies have demonstrated significant inaccuracies in the diagnosis, a finding that has been previously documented [116,117]. Manual palpation and diagnostic criteria according to Travell and Simons currently constitute the standard examination [4,26], and the majority of the studies (20) explicitly referenced them. In their respective works, several authors documented the presence of a “painful spot, referred pain, or taut band”. However, only six of the authors mentioned “recognition of pain”, which Simons considers the primary diagnostic criterion [4,26,27]. It is noteworthy that the precise distinction between active and latent MTrP was present in only five studies [80,95,102,113,114]. In contrast, nine authors reported only the identification of a trigger point, while seven authors did not report any of the criteria mentioned above and thus lacked accuracy regarding the initial diagnosis. Moreover, the majority of studies were conducted on MPS in the upper trapezius muscle (UTM), and the present data set lacks comprehensive information regarding the number of MTrPs identified during examination. The existence of multiple MTrPs in MPS of neck and shoulder muscles, including UTM, has been documented in the literature [118,119]. Notably, several studies reported the presence of only a single MTrP in the UTM.

Furthermore, it is essential to highlight the striking absence of information regarding the examiners’ experience during the examination process. Only six studies included information regarding the examiner’s experience [85,102,103,106,110,114]. Twenty-one studies did not provide any information regarding the qualifications of the examiners. It has been observed in the literature that the quality of MTrP examination is significantly influenced by the experience of the examiner [26,27,28,120,121].

Furthermore, only a limited number of studies provided information regarding the background and experience of the operator performing the intervention, without offering precise data regarding their qualifications, such as a certificate and the number of years of shockwave treatment experience. It is of significant importance to consider the experience and qualifications of the practitioner when administering ESWT. Moreover, the International Society for Medical Shockwave Treatment (ISMST) has recommended in their Consensus Statement from 2022 that only a qualified physician (certified by a national or international medical society) may utilize fESWT.

It is also notable that the treatment protocols demonstrated considerable heterogeneity. For example, the parameters for extracorporeal shockwave therapy (ESWT) varied widely, with a dose range of 1.2–4 bar or 0.056–0.38 mJ/mm^2^. Furthermore, the number of impulses applied to MTrPs exhibited a considerable range, varying from 300 to 1500. The frequency of the emitted pulses, specified in hertz, also demonstrated considerable variation, spanning from 1 to 20 hertz. Notably, 12 studies failed to mention this parameter. The frequency is regarded as an essential aspect of the application of fESWT [8,122].

It is also noteworthy that in some studies, a considerable amount of energy was used, while in others, the energy expenditure was minimal. This pattern also applies to the number of therapy sessions, which are indicated in the study protocols with extremes of 1 and 24. It is crucial to highlight that these extremes are not in accordance with the ISMST guidelines and are not stated in any of the protocols of the other studies. As previously stated, the energies of RPW, which is physically accurately represented in bar, and ESWT, which is measured in EFD (mJ/mm^2^), are distinct. However, in one-third (8 of 24) of the studies in which RPW was utilized, the energy was stated incorrectly in EFD. This inaccuracy was not addressed in the meta-analyses. This incorrect designation could indicate a lack of experience with the use of shockwaves. Furthermore, the assumption that the lack of knowledge of the users could have played a role in the results of the studies is also confirmed by the fact that the qualifications and experience were only very inadequately stated (see above).

A further issue is the absence of documentation regarding the precise application of shockwaves on the trigger point (MTrP), which the patient’s feedback would indicate. The precision of MTrP treatment plays a significant role in the efficacy of myofascial pain treatment [9,23,123,124,125,126]. The literature describes the advantages of using focused ESWT for diagnosing myofascial pain and triggering diagnostic criteria [127]. Regrettably, only a limited number of studies documented the precise identification of the MTrPs and the exact application, which would be determined by the diagnostic criteria [4,22,23,26,27,80,120,121]. Furthermore, due to the lack of an accurate description of ESWT application, it can be assumed that the same standards were not adhered to.

A further issue with the selected studies for the meta-analysis is the disparate manner in which the timing of the follow-up was chosen across the available studies. The interval between the final treatment and the follow-up examination ranged from 0 to 15 weeks, with many studies having only a short follow-up of 2 weeks or less (12 of 36). This is of particular significance given that numerous mechanisms of action, as previously outlined, necessitate a more extended period of action. Therefore, the complete efficacy of this therapeutic approach could not be ascertained within the confines of a brief follow-up interval. It can be postulated that a more extended follow-up period, similar to that employed in the ESWT studies, which successfully demonstrated the impact of ESWT on a multitude of musculoskeletal disorders [8,17,18], would have yielded disparate outcomes. This fact is also reflected in the results of some studies, in which it was demonstrated that a notable therapeutic outcome was not yet apparent in the follow-up examination conducted after one or two weeks. However, this outcome became evident 4 weeks or 12 weeks later [92,94]. In addition, studies on ESWT with a shorter study period than 4 weeks, preferably 12 weeks, should be excluded from the evaluations. The collective findings of these reviews and meta-analyses demonstrated the efficacy and safety of myofascial ESWT in the treatment of musculoskeletal pain, with generally comparable and consistent results. It is noteworthy that the ESWT intervention yielded only a significant improvement in pain relative to placebo ESWT or ultrasound. This improvement does not reach a level that is superior to that observed with other conventional modalities, such as TPI, dry needling, or laser therapy.

## 5. Conclusions

In light of these considerations, it is essential to exercise caution when interpreting the results of the meta-analyses, as significant shortcomings were identified in numerous individual studies included. These shortcomings are particularly evident in the inconsistent diagnosis of MTrP, the markedly diverse types of shockwave application, and the substantially disparate follow-up periods. Furthermore, some treatment protocols seemed to be inadequate, which did not align with previous treatment procedures or the ISMST guidelines. It is also noteworthy that meaningful subgroups are formed in reviews, particularly concerning RPW and fESWT. It is recommended that studies on ESWT with a shorter study period than 4 weeks, preferably 12 weeks, be excluded from the evaluations.

A further limitation of the meta-analyses is the imprecise integration of the RPW and fESWT therapeutic modalities. Although these two procedures utilize two distinct physical energies, they are erroneously grouped in the meta-analyses under the term ESWT (extracorporeal shockwave therapy). These two methods must not be conflated in the analysis or the presentation of the studies. Additionally, in subsequent publications, both methods should be clearly referred to separately as radial pressure wave (RPW) and focused Extracorporeal Shockwave Therapy (fESWT).

Currently, there are no standardized imaging techniques to support the diagnosis of myofascial syndrome. As long as no examination technique for myofascial trigger points provides reliable, reproducible diagnostic data, research in this area will remain on scientifically unstable ground. This highlights the importance of using established clinical parameters, such as diagnostic criteria, in studies of myofascial pain. These criteria can be directly determined by using focused extracorporeal shockwave therapy (fESWT) to diagnose myofascial pain, as previously described in the literature.

In conclusion, myofascial ESWT has recently attracted considerable interest within the medical community, as evidenced by the numerous studies and meta-analyses that have been conducted in this field. However, in the absence of standardized study protocols, it is of the utmost importance to obtain new scientific data from clinical research to advance this non-invasive treatment modality.

Future studies of myofascial ESWT should include quality standards for shockwave therapy in terms of extended follow-up, description of the application, qualifications (certificate) and experience of the practitioner, and the principles of diagnosis of myofascial syndrome, particularly diagnostic criteria. It is recommended that future studies employ the guidelines set forth by the International Society of Medical Shockwave Treatment (ISMST) as a framework (https://shockwavetherapy.org/ismst-guidelines/, accessed on 14 September 2025), as they represent a synthesis of previous study protocols, therapeutic principles, and the collective experience of best practice over the past two decades. Any deviations from these guidelines should be clearly documented in the study protocol.

## Appendix A

**Table A1:** Summary of the studies for diagnosis and characteristics of the intervention.

Reference	Study Design	Sample Size	Examiner Experience		Operator Experience with ESWT		Diagnostic Criteria of MPS	Diagnostic Criteria by Manual Palpation	Region of Application	Targeted Muscle
			Years	Qualification	Years	Qualification				
**Ali. 2016** [82]	RCT	30	n.m.	n.m.	n.m.	n.m.	n.m.	TrP	shoulder	Rot cuff
**Anwar 2022** [83]	RCT	45	n.m.	Physiatrist	n.m.	Physiatrist	TrP	TrP	neck, shoulder	Up Trap
**Aktürk 2018** [84]	RCT	30	n.m.	Medical Doctor	n.m.	Physiatrist	Trav-Simons	n.m.	n.m.	n.m.
**Carlisi 2021** [85]	RCT	55	>5	Rehab Specialist	>5	Rehab Specialist	Trav Simons	n.m.	calf, foot	Gastroc, soleus
**Cho 2012** [86]	RCT	36	n.m.	Orthopeadic Specialist	n.m.	n.m.	n.m.	n.m.	neck, shoulder	Up Trap
**Damian 2011** [87]	RCT	26	n.m.	n.m.	n.m.	Physiotherapist	n.m.	TrP	neck, shoulder, head, TMJ	Temp, Mass, Trap, SCM, rhomb
**Elhafez 2021** [89]	RCT	60	n.m.	n.m.	n.m.	n.m.	n.m.	TB, TrP, Ref, Rec	neck, shoulder	Up Trap
**Elhafez 2022** [88]	RCT	60	n.m.	n.m.	n.m.	n.m.	n.m.	TrP, LT	neck, shoulder	Up Trap
**Eftekharsadat 2020** [90]	RCT	54	n.m.	n.m.	n.m.	n.m.	Trav Simons	TB, TrP, Ref, LT	low back	Quad Lumb
**Gezginaslan 2020** [91]	RCT	94	n.m.	n.m.	n.m.	n.m.	Trav Simons	TrP	neck, shoulder	Up Trap, infra, supra
**Gur 2013** [92]	RCT	60	n.m.	Medical Doctor	n.m.	Physiotherapist	Trav Simons	TrP	neck, shoulder	Up Trap
**Hong 2017** [93]	Retrospective study	30	n.m.	n.m.	n.m.	n.m.	Trav Simons	TB, TrP, Ref	lower back,	Quad Lomb
**Huang 2014** [94]	RCT	90	n.m.	n.m.	n.m.	n.m.	n.m.	n.m.	neck, shoulder, lower back	n.m.
**Ibrahim 2017** [95]	RCT	30	n.m.	n.m.	n.m.	n.m.	Trav Simons	TB, TrP, Ref	cervical	Up Trap
**Jeon 2012** [80]	RCT	30	n.m.	n.m.	n.m.	n.m.	n.m.	TB, TrP, Ref, Rec	neck, shoulder	Up Trap
**Ji 2012** [96]	RCT	20	n.m.	Medical Doctor	n.m.	Medical Doctor	Trav Simons	TB, TrP, Ref	neck, shoulder	Up Trap
**Kamel 2020** [97]	RCT	46	n.m.	Orthopeadic Specialist	n.m.	n.m.	Trav Simons	TB, TrP, Ref	neck	Up Trap
**Kiraly 2018** [98]	RCT	61	n.m.	n.m.	n.m.	n.m.	Trav Simons	TB, TrP, Ref	neck, shoulder	Up Trap
**Lee 2012** [99]	RCT	31	n.m.	n.m.	n.m.	n.m.	Trav Simons	TB, Ref	neck, shoulder	Up Trap
**Lee and Han 2013** [100]	RCT	33	n.m.	n.m.	n.m.	n.m.	Trav Simons	n.m.	neck, shoulder	Up Trap
**Li 2020** [101]	RCT	80	n.m.	n.m.	n.m.	n.m.	n.m.	n.m.	TMJ	n.m.
**Luan 2019** [102]	RCT	65	20	Clinician	n.m.	Physiotherapist	Trav Simons	TB, TrP, Ref, Rec, LT	neck, shoulder	Up Trap
**Manafnezhad 2019** [103]	RCT	70	5	Physiotherapist	5	Physiotherapist	Trav Simons	TB, TrP,	neck, shoulder	Up Trap
**Moghtaderi 2014** [104]	RCT	40	n.m.	n.m.	n.m.	n.m.	n.m.	TrP	foot, calf	Gastroc, soleus
**Mohamed 2021** [105]	RCT	60	n.m.	n.m.	n.m.	n.m.	n.m.	TB, TrP	neck, shoulder	Up Trap
**Park 2018** [106]	RCT	30	3	Physiatrist	>3	Physiatrist	Trav Simons	TB, TrP, Ref, LT	neck, shoulder	Up Trap
**Rahbar 2021** [107]	RCT	48	n.m.	Physiatrist	n.m.	Physiatrist	n.m.	TB, TrP, Ref	neck, shoulder	Up Trap, Mid Trap, lev scap, rot cuff
**Sukareechai 2019** [108]	RCT	42	n.m.	n.m.	n.m.	n.m.	Trav Simons	TB, TrP, Ref, Rec	neck, shoulder	Up Trap, infra, rhomb
**Sugawara 2021** [109]	Retrospective study	1580	n.m.	Physiatrist	n.m.	Physician	n.m.	n.m.	limbs	n.m.
**Suputtitada 2022** [110]	RCT	60	>5	Physiatrist	n.m.	n.m.	Trav Simons	TrP	neck, shoulder	Up Trap
**Taheri 2016** [111]	RCT	46	n.m.	n.m.	n.m.	n.m.	Trav Simons	TB, TrP, Rec	neck, shoulder	Up Trap
**Taheri 2021** [112]	RCT	37	n.m.	Physician	n.m.	Therapist	n.m.	TrP	neck, shoulder	Up Trap
**Toghtamesh 2021** [113]	RCT	16	n.m.	Experienced Therapist	n.m.	n.m	n.m.	TB, TrP, Ref, Rec	neck, shoulder	Up Trap
**Walsh 2019** [114]	RCT	21	3	Athletic Therapist	2	Athletic Therapist	Trav Simons	TB, TrP, latent TrP	lower limb	Vast Med, Vast Lat
**Yalcin 2021** [115]	Retrospective study	262	n.m.	Physician	n.m.	Physician	Trav Simons	TrP	neck, upper back	Up Trap

Abbreviations: n.m. = not mentioned; TB = Taut Band; TrP = myofascial Trigger Point; Ref = referral of pain; LT = local twitch response; Rec = recognition of pain; Up Trap = M. upper trapezius; Mid Trap = M. trapezius middle part; lev scap = M. levator scapu.

## Appendix B

**Table A2:** Summary of the protocol characteristics and parameters.

Reference	Shockwave Device	Sessions	Interval Between Sessions (days)	Energy	Frequence (Hz)	Impulses/TrP	Impulses/session	Total Impulses	Diagnostic Criteria by Shockwave Application	Follow-up (Weeks After Last Session)
**Ali. 2016** [82]	Radial	3	7	0.38 mJ/mm^2^; 1.6 bar	10	<500	2000	6000	TrP	4
**Anwar 2022** [83]	Radial	3	7	1.2 bar	5	1000	1000	3000	n.m.	1 and 4
**Aktürk 2018** [84]	Radial	4	3	6 bar	4	200–400	2000–3000	8000–12,000	n.m.	6
**Carlisi 2021** [85]	Focused	3	7	0.15 mJ/mm^2^	8	400	1200–1700	8700	n.m.	8
**Cho 2012** [86]	Radial	12	2	0.12 bar	n.m.	1000	1000	12,000	n.m.	0
**Damian 2011** [87]	Radial	5–6	7	n.m.	n.m.	n.m.	n.m.	n.m.	n.m.	0
**Elhafez 2021** [89]	Radial	4	7	1.5 bar	1	300	300	1200	TrP	4
**Elhafez 2022** [88]	Radial	4	3	0.056 mJ/mm^2^	10	400	700	2800	TrP	0
**Eftekharsadat 2020** [90]	Radial	5	7	0.1 mJ/mm^2^	10–16	1500	1500	7500	n.m.	4
**Gezginaslan 2020** [91]	Radial	7	3	1.5–3 bar	n.m.	500	1500–4500	10,500–44,500	TrP	4
**Gur 2013** [92]	Focused	3	3	0.25 mJ/mm^2^	n.m.	n.m.	1000	3000	TrP	3 and 12
**Hong 2017** [93]	Focused	3	3	0.085–0.148 mJ/mm^2^	n.m.	2000	2000	6000	TrP	4
**Huang 2014** [94]	Focused	30	3	0.18–0.25 mJ/mm^2^	1	700	n.m	n.m.	TrP, Ref	2.4 and 12
**Ibrahim 2017** [95]	Radial	4	3	2.0–2.6 bar	4–20	1000	2000	8000	TrP	4
**Jeon 2012** [80]	Focused	3	7	0.10 mJ/mm^2^	n.m.	n.m.	1500	4500	TrP, Ref, LT	0
**Ji 2012** [96]	Focused	4	3	0.056 mJ/mm^2^	n.m.	700	1000	4000	TB	0
**Kamel 2020** [97]	Focused	4	7	0.25 mJ/mm^2^	n.m.	1000	1000	4000	TrP	4
**Kiraly 2018** [98]	Radial	3	7	1.5–2.5 bar	10	1000	2000	6000	n.m.	3 and 15
**Lee 2012** [99]	Radial	2	n.m.	n.m.	5	n.m	800	1600	n.m.	0
**Lee and Han 2013** [100]	Radial	8	4	n.m.	5	1000	1000	1000	TrP	n.m.
**Li 2020** [101]	Radial	4	7	n.m.	8	n.m.	1000–1500	4000–6000	n.m.	4
**Luan 2019** [102]	Radial	3	7	0.10 bar	n.m.	1500	2000	6000	TrP, LT	4 and 12
**Manafnezhad 2019** [103]	Radial	3	1	60 mJ	16	1000	1000	3000	n.m.	1
**Moghtaderi 2014** [104]	Focused	3	2	0.2 mJ/mm^2^	n.m.	400	>3400	>10,200	TrP	8
**Mohamed 2021** [105]	Radial	4	7	1.5 bar	8	1000	1000	4000	n.m.	4
**Park 2018** [106]	Focused	2	7	0.068–0.21 mJ/mm^2^	n.m.	1500	1500	3000	Ref, LT	2
**Rahbar 2021** [107]	Radial	4	7	60 mJ/m^2^	5	500–2000	2000	8000	TrP	1 and 4
**Sukareechai 2019** [108]	Radial	3	7	1–2 bar	12	300	up to 6000	up to 18,000	TrP	0
**Sugawara 2021** [109]	Radial	2	7	variable	variable	variable	variable	variable	n.m.	1
**Suputtitada 2022** [110]	Radial	3	7	2.5 bar	12	2000	2000	6000	TrP	1
**Taheri 2016** [111]	Focused	3	7	0.003 mJ/mm^2^	10	n.m.	1000	3000	TrP	4
**Taheri 2021** [112]	Focused	3	7	0.2 mJ/mm^2^	10	n.m.	2000	6000	TrP	4
**Toghtamesh 2021** [113]	Radial	1	0	0.038 mJ/mm^2^	10	700	1000	1000	TB, TrP	0
**Walsh 2019** [114]	Radial	3	2	up to 5 bar	20	2000	3000	9000	TrP	1
**Yalcin 2021** [115]	Radial	3	7	0.056 mJ/mm^2^	n.m.	1500	1500	4500	TrP	12

Abbreviations: n.m. = not mentioned; TB = Taut Band; TrP = myofascial Trigger Point; Ref = referral of pain; LT = local twitch response.
